# Special Issue “Biomolecular Structure, Function and Interactions”

**DOI:** 10.3390/ijms262311731

**Published:** 2025-12-04

**Authors:** Ivo Crnolatac

**Affiliations:** Division of Organic Chemistry and Biochemistry, Ruđer Bošković Institute, Bijenička 54, 10000 Zagreb, Croatia; icrnolat@irb.hr

Welcome to the Special Issue of *International Journal of Molecular Sciences*, titled “Biomolecular Structure, Function, and Interactions.” The ten original research contributions and one review gathered here reflect a rapidly evolving field in which experimental, computational, and machine learning methodologies converge to provide deeper insights into how the sequence, structure and dynamics of biomolecules give rise to function, and how those functions shape interactions in biological systems.

The classical idea in molecular life sciences states that the linear amino acid sequence dictates the folded three-dimensional structure, which then determines the biological function of the protein. This sequence–structure–function system is a foundation; however, we now have two parallel highways that are reshaping the way we examine this axis. Advances in structural biology (X-ray crystallography, NMR, cryo-EM, single-molecule spectroscopy) continue to provide ever higher-resolution snapshots of biomolecular machines, their complexes, and conformational assemblies. On the other hand, the explosion of sequence data (e.g., UniProt now lists hundreds of millions of sequences) together with algorithmic and hardware advances in machine learning and artificial intelligence (AI) have rendered powerful data-driven models of structure, dynamics, and interaction [1,2]. Structural modelling has been transformed by AI (e.g., the success of deep neural-networks in structure prediction), and models of interaction (protein–protein, protein–small molecule) have followed suit. In this context, the contributions in this Special Issue illustrate three major trends:

*Integration of experimental and computational methods*—structures that were derived empirically provide scaffolds for modelling, while computational predictions guide experiment.

*Machine-learning-driven abstraction*—the embedding of molecular features (sequence, structure, dynamic descriptors) into predictive frameworks.

*Function*—an emergent property of interactions and dynamics, not a static endpoint of structure alone.

## 1. Highlights of This Issue

López-Cortés et al. in Contribution 1 curate chemical descriptors (for ligands), protein sequence/structure features (for odorant/pheromone-binding proteins), and functional assay data (ligand-binding responses) to then build regression models. This work appears to be the first to build a quantitative bridge across these three data types in the insect olfaction context.

The authors Gálvez-Ramírez et al. (Contribution 2) combine biochemical assays with in silico structural modelling to elucidate the molecular basis of three class A G6PD variants—a more classical structure–function inquiry still enriched with computational tools.

Shih and Hsu (Contribution 3) demonstrate how an engineered peptide can stabilize a protein of biomedical relevance, illustrating an interface of structure, function, and therapeutic potential.

Filić et al. (Contribution 4) use site-directed mutagenesis, enzymatic assays, and computational modelling to detail how conserved residues mediate multifunctionality in an industrially relevant enzyme, showing the enduring importance of classic biochemistry when accompanied with in silico insight.

A deep learning approach for understanding structure and consequently the function of *Chlorella vulgaris* hydrogenase is presented by Botticelli et al (Contribution 5).

Desai et al. (Contribution 6) used the Deep Learning Model for Transmembrane Topology Prediction and Classification (Deep TMHMM) [3] combined with UniProt to tackle the experimentally challenging field of membrane proteins.

The review by Bui and Inaba (Contribution 7) offers a comprehensive view of transporter structure–mechanism–function relationships, highlighting how conformational transitions, metal binding, and topology intersect in homeostasis.

Additional contributions (8 and 9) (e.g., “Genome-wide analysis of AUX/IAA gene families, structural insights into PBP4 from M. tuberculosis”, “Cortical and Striatal Astrocytes of Neonatal Rats Display Distinct Molecular and Pharmacological Characteristics of Dopamine Uptake”) round out a rich mixture of methodological and thematic diversity. These works demonstrate not just isolated advances but underline an emergent methodological system: high-throughput sequencing, machine-learning embedding or regression, structure prediction/docking, experimental validation, and ultimately function or interaction assay.

## 2. Outlook and Future Directions

Looking ahead, several themes merit emphasis for our research area:

From static to dynamic: While structure prediction (via AI) has achieved remarkable fidelity, capturing dynamics, ensembles and transient interactions remain a challenge. The integration of time-resolved experimental data (e.g., single-molecule, cryo-EM of flexible complexes) with machine learning will become increasingly important [4].

Interpretable machine learning (ML) and mechanistic insight: ML models can provide strong predictive performance, but mechanistic interpretability is often limited. We seek meaningful mechanistic explanations and insights. Prediction alone, no matter how precise, is not enough. The newly emerged area of explainable artificial intelligence (XAI) tries to cover this gap [5]. Hybrid approaches that embed physical constraints or known biochemical mechanisms (rather than treating ML as a “black box”) will likely dominate the next wave of impactful contributions.

Deep integration of ligand/chemical space, protein sequence/structure, and functional output: The work by López-Cortés et al. (Contribution 1) in this Special Issue is a promising leap in that direction; we anticipate more studies that integrate all three modalities.

Expanding the interaction universe: Beyond protein–protein and protein–ligand interactions, interactions with nucleic acids, membranes, small metabolites, and the cell-microenvironment will be increasingly modelled using integrative pipelines. The recent “AI meets biomolecular interaction prediction” review [6] attests this trend.

Of course, the future will not only involve decoding natural biomolecular phenomena but also designing novel biomolecules (enzymes, binders, sensors) and translating structure–function knowledge into therapeutic or biotechnological applications.

## 3. Concluding Remarks

This Special Issue is timely because it brings together examples of how experimental rigour, computational sophistication, and machine learning innovation can be interwoven into coherent investigative threads. The mesh of these approaches represents not a sum of parts, but rather a synergistic methodology now accessible to many researchers.

I invite you to take a system-level view while reading these contributions: consider how sequence, structure, ligand, descriptor, model, and assay all interconnect, and how the methodologies themselves are evolving. I hope this Special Issue not only showcases current achievements but also stimulates new collaborations across disciplines (structural biology, data science, computational chemistry, bioengineering) and encourages the development of integrative workflows you may adopt or adapt in your own research.

I thank all authors for their excellent contributions, the reviewers for their insightful feedback, and the editorial office of *IJMS* for their support. I believe this Special Issue will serve as a valuable resource for scientists interested in the frontiers of biomolecular structure–function–interaction, and that it stimulates further advances in this rich and expanding area of research, with best wishes for your explorations of the structure, function, and interaction of biomacromolecules.
